# Health Care Costs Associated to Type of Feeding in the First Year of Life

**DOI:** 10.3390/ijerph17134719

**Published:** 2020-06-30

**Authors:** Carolina Lechosa-Muñiz, María Paz-Zulueta, María Sáez de Adana Herrero, Elsa Cornejo del Rio, Sonia Mateo Sota, Javier Llorca, María J. Cabero-Perez

**Affiliations:** 1 Breastfeeding Coordinator, IBCLC, Hospital Universitario Marqués de Valdecilla, 39008 Santander, Spain; carolina.lechosa@scsalud.es; 2 Faculty of Nursing, Universidad de Cantabria, 39008 Santander, Spain; 3 IDIVAL, GI Derecho Sanitario y Bioética, GRIDES, 39008 Santander, Spain; 4 Supervisor, Gynecology Service, Hospital Universitario Marqués de Valdecilla, 39008 Santander, Spain; maria.saezdeadana@scsalud.es; 5 Obstetrics Service, Hospital Universitario Marqués de Valdecilla, 39008 Santander, Spain; elsacdelrio@gmail.com; 6 Pediatrics Service, Hospital Universitario Marqués de Valdecilla, 39008 Santander, Spain; soniamsota@yahoo.es (S.M.S.); mariajesuscabero@gmail.com (M.J.C.-P.); 7 Department of Medical and Surgical Sciences, Universidad de Cantabria, 39008 Santander, Spain; javier.llorca@unican.es; 8 IDIVAL, GI Epidemiología y Salud Pública, 39008 Santander, Spain; 9 CIBER Epidemiology and Public Health (CIBERESP), 28029 Madrid, Spain; 10 IDIVAL, Universidad de Cantabria, 39008 Santander, Spain

**Keywords:** breastfeeding, cost of illness, artificial feeding, economic evaluation

## Abstract

Background: Breastfeeding is associated with lower risk of infectious diseases, leading to fewer hospital admissions and pediatrician consultations. It is cost saving for the health care system, however, it is not usually estimated from actual cohorts but via simulation studies. Methods: A cohort of 970 children was followed-up for twelve months. Data on mother characteristics, pregnancy, delivery and neonate characteristics were obtained from medical records. The type of neonate feeding at discharge, 2, 4, 6, 9 and 12 months of life was reported by the mothers. Infectious diseases diagnosed in the first year of life, hospital admissions, primary care and emergency room consultations and drug treatments were obtained from neonate medical records. Health care costs were attributed using public prices and All Patients Refined–Diagnosis Related Groups (APR–DRG) classification. Results: Health care costs in the first year of life were higher in children artificially fed than in those breastfed (1339.5€, 95% confidence interval (CI): 903.0–1775.0 for artificially fed vs. 443.5€, 95% CI: 193.7–694.0 for breastfed). The breakdown of costs also shows differences in primary care consultations (295.7€ for formula fed children vs. 197.9€ for breastfed children), emergency room consultations (260.1€ for artificially fed children vs. 196.2€ for breastfed children) and hospital admissions (791.6€ for artificially fed children vs. 86.9€ for breastfed children). Conclusions: Children artificially fed brought about more health care costs related to infectious diseases than those exclusively breastfed or mixed breastfed. Excess costs were caused in hospital admissions, primary care consultations, emergency room consultations and drug consumption.

## 1. Introduction

Breastfeeding is one of the most efficacious tools for preventing diseases and for promoting health in both mothers and children [1,2,3,4]. The World Health Organization recommends: “exclusive breastfeeding for the first six months and breastfeeding complemented until two years or more” [5]. Global costs of not breastfeeding have been estimated as about $302 billion [6] and local/state studies have also provided cost saving estimates of breastfeeding [7]. Investment in deeds favoring breastfeeding onset and continuation has been proved to be not only healthy but also cost saving [8,9,10,11].

The benefits of breastfeeding on maternal and neonatal health result in lower demand for health services in both primary and specialized care, decreasing the number of hospital admissions and drug treatments [12,13,14,15,16,17]. Regarding infectious diseases, breastfeeding has been associated with lower risk of, especially, diarrhea and pneumonia, but also bronchiolitis and otitis, although this association is less consistent in high-income countries [18]. On the other hand, children artificially fed suffer higher morbidity and mortality, eventually leading to higher social and economic costs [19,20,21,22,23,24].

As no cohort study has been carried out in Spain regarding costs associated to type of feeding, the aim of this study is to calculate the economic repercussions of breastfeeding via decreasing infectious disease incidence in the first year of life. To this purpose, we followed-up a cohort of consecutive neonates in Cantabria, North of Spain.

## 2. Methods

### 2.1. Participants and Recruitment

We carried out a cohort study by recruiting 970 consecutive neonates in the University Hospital Marqués de Valdecilla (HUMV), Santander, Spain, from 1 January 2018, on. The HUMV is a public hospital part of the Cantabria Health System (Servicio Cántabro de Salud, (SCS)). It attends about 3000 deliveries per year. Details on design and recruitment have been published elsewhere [25]; this manuscript is a further analysis of that sample after following children for one year.

### 2.2. Information

Medical records of both mothers and neonates were reviewed in order to gather information on maternal age, educational level, occupational situation and smoking habits. Regarding neonate information, we recorded gestational age, birth order and nursery attendance. Type of feeding (exclusive breast feeding, mixed and exclusive artificial feeding) was recorded at hospital discharge and at 2, 4, 6, 9 and 12 months of life. It was considered that WHO’s definition for “exclusive breastfeeding” is defined as no other food or drink, not even water, except breast milk (including milk expressed or from a wet nurse) for 6 months of life, but allows the infant to receive ORS, drops and syrups (vitamins, minerals and medicines) since birth [26].

### 2.3. Follow-Up

To estimate health care costs associated to type of feeding via infectious diseases, we recorded each infectious disease occurring in the first 12 months and each health care system utilization due to those infectious diseases in the first year of life. These included number of consultations with primary care pediatricians, number of consultations with hospital pediatricians, drug treatment, lab tests, number of visits to emergency room and number of hospital admissions.

### 2.4. Cost Estimation

Only direct health care costs were estimated from the health service perspective. Costs of hospital admissions were estimated using the All Patients Refined–Diagnosis Related Groups (APR–DRGs) version 35 [27]. Costs of primary care consultations, emergency room and drug treatment were obtained from the SCS public prices [28]. From here on, costs refer to the aggregated cost in the first 12 months of life due to infectious diseases.

### 2.5. Statistical Analysis

Results are expressed as mean ± standard deviation for continuous variables and number (percentage) for categorical variables. The relationships between the type of newborn feeding and health care costs were analyzed using multiple linear regression. We carried out a regression analysis for each type of cost as an outcome, introducing type of feeding as a categorical regressor; all regression models were adjusted for maternal smoking, maternal educational level, maternal occupational status, twin pregnancy, gestation length, birth order, nursery attendance (yes/no) and age of starting nursery attendance. Its results are presented as marginal means in euros with 95% confidence intervals (CI). Regarding interpretation of marginal means, let us suppose results were 200, 250 and 300€ for exclusive breastfeeding, mixed and artificial feeding, respectively. This would mean that if the whole sample had been exclusively breastfed, the average cost would have been 200€; if the whole sample had been mixed fed, the average cost would have been 250€ and if the whole sample had been artificially fed, the average cost would have been 300€.

### 2.6. Ethical Considerations

This project was approved by the Ethics Committee for Clinical Research of Cantabria in July 2017 (Ethical approval code 2017.142). The parents signed an informed consent for participating in the study. The project was carried out according to the Spanish laws on biomedical research, the European Union regulations on the protection of natural persons with regard to the processing of personal data and the Declaration of Helsinki on ethical principles for medical research involving human subjects.

## 3. Results

The cohort recruited 970 neonates born from 948 women. Average maternal age was 33.7 ± 5.2 years, 36.9% women had university studies, 69.6% were workers and 12.5% were smokers. Pregnancy length was 39.1 ± 2.0 weeks. Only 6.2% births were preterm (i.e., born before week 37), and 8.7% neonates weighed less than 2500 g. At hospital discharge, 54.0% of neonates were breastfed, 28.0% were fed with mixed breastfeeding and artificial and 17.9% were fed only artificially (Table 1).

### 3.1. Neonate Type of Feeding and Consultations in Primary Care

Neonates with exclusive breastfeeding at hospital discharge (*n* = 524) consulted 1217 times in primary care due to infectious diseases in their first year of life (ratio = 2.3), which costed 197.9€/neonate on average (95% CI: 177.0–218.8). Neonates with artificial feeding (*n* = 174) consulted 683 times in primary care (ratio = 3.9), costing on average 295.7€ (95% CI: 258.5–332.8) (Table 2). Neonates fed with mixed natural + artificial feeding had intermediate values (number of consultations/number of neonates ratio = 2.7; average cost: 223.2€, 95% CI: 194.8–251.6). As time went by, some mothers abandoned exclusive breastfeeding, so the number of neonates with mixed or artificial feeding increased. Then differences in costs generated in primary care consultations decreased as shown in Table 2.

### 3.2. Neonate Type of Feeding and Consultations in Emergency Room

Ratios of number of consultations in emergency room/number of neonates according to type of feeding at hospital discharge were 1.0 for breastfeeding, 1.3 for mixed feeding and 1.6 for artificial feeding. Costs associated to consultations in emergency room were higher in neonates with artificial feeding at hospital discharge than in neonates breastfed (260.1€, 95% CI: 206.7–313.6 in artificially fed and 196.2€, 95% CI: 165.3–227.0 in breastfed neonates, *p* = 0.05), with neonates fed with mixed generating midway costs. When studying costs associated with type of feeding at two, four and six months of life, differences remained about the same (Table 3).

### 3.3. Neonate Type of Feeding and Admissions for Infectious Disease

Admissions to hospital due to infectious diseases are described in Supplementary Table 1, including APR–DRG code, APR–DRG description, disease severity, DRG weight according to Spanish rules, normalized cost in €/patient, number of patients and total cost for each APR–DRG. There were 39 admissions in the first year of life, with respiratory syncytial virus pneumonia being the most frequent cause (*n* = 10). According to feeding at hospital discharge, the quotient number of admissions/number of neonates was 1.3% for breastfed neonates, 6.3% for mixed fed neonates and 8.6% for artificially fed neonates (Table 4). Average costs per neonate were 86.9€ in breastfed neonates and 791.6€ per artificially fed neonate (Table 4).

### 3.4. Neonate Type of Feeding and Health Care Costs

Table 5 summarizes health care costs associated to infectious diseases in the first year of life; this is the result of adding costs due to primary care consultations (Table 2), emergency room consultations (Table 3), admissions to hospital (Table 4) and treatment with drugs (Supplementary Table 2). When considering type of feeding at hospital discharge, neonates breastfed and those fed with mixed produced similar costs (443.5€, 95% CI: 193.7–694.0 for breastfeeding vs. 571.4€, 95% CI: 235.0–907.8 for mixed feeding; *p* = 0.56). Neonates fed with artificial feeding, however, used more resources of the health care system due to infectious diseases (1339.5€, 95% CI: 903.0–1775.0, *p* = 0.001).

## 4. Discussion

According to our results in a cohort of 970 infants, neonates fed with artificial feeding at hospital discharge use more resources of the health care system due to infectious diseases: they have more consultations in primary care and emergency room, more admissions to hospital and produce more health care costs than neonates exclusively or partially breastfed. At the end of their first year of life, their health care cost associated to infectious diseases is about 900€ higher than that of exclusively breastfed infants. This result is higher than that previously reported by Santacruz-Salas et al. in a smaller Spanish cohort [29], although they compared exclusive vs. non-exclusive breastfeeding until six months, while we have separated the last group into mixed breastfeeding + artificial feeding and only artificial feeding. Taking into account that 18% of neonates in our cohort were fed with artificial feeding, if our study could be extrapolated to the 373,000 newborns in Spain in 2018, it could result in an excess health care cost of about 60.5 million Euro. In the Spanish public health system, costs of hospital admission or emergency room/primary care consultations are fully covered by the health system, while drug costs are partially supported by the parents. Therefore, most of the excess costs attributed to artificial feeding founded in our analysis is funded with taxes.

International figures are hard to compare as unit costs would be different from country to country. However, when Pokhrel et al. [16] carried out a simulation on costs saved in the United Kingdom by exclusive breastfeeding in only five diseases (gastrointestinal illness, acute otitis media, lower respiratory tract infection, necrotizing enterocolitis and breast cancer), they used £1078 as a baseline cost for hospital admission due to lower respiratory tract infection, which is not far from the Spanish cost estimate (1856€ per case in APR–DRG number 240—non-bacterial gastroenteritis, nausea and vomiting, severity 1, Table S1). Pokhrel et al. results stated that exclusive breastfeeding for four months would have saved £11 million per year [16]. Walters et al. developed a tool for estimating costs attributable to suboptimal breastfeeding [24]. They estimated global health care costs due to childhood diarrhea to be $196.19 million and costs due to childhood pneumonia to be $696.69 million [24]. It is noteworthy that both Pokhrel et al. and Walters et al. were simulation studies, not actual cohorts as our study is.

As shown in Table 2, Table 3, Table 4 and Table 5, health care cost differences between types of feeding tend to decrease when type of feeding is determined at 2, 4, 6, 9 and 12 months.

Our study has some limitations. Firstly, our study is based on information gathered from medical records. Therefore, its quality is conditioned by exhaustivity and reliability of records, which could not be tested. In order to minimize this problem, we chose variables usually recorded in a systematic and objective way in electronic medical records. We presume non-recorded data would equally affect children, whatsoever their type of feeding; therefore, we conjecture that the differences in costs we have found could be scarcely affected by this limitation. Secondly, our study is observational in nature and causal relationships cannot be established. In this regard, we cannot rule out that our results could have been due to unmeasured confounding factors. The health care cost difference we have found between exclusive breastfeeding and artificial feeding is, however, so important that a confounder able to explain it should have a very strong relationship with both type of feeding and health care costs, which makes such a confounding factor unlikely. Thirdly, we have attributed health care costs in the first year of life to types of breastfeeding as recorded in different times (at discharge, 2, 4, 6, 9 and 12 months). Temporal precedency can only be stated for feeding at discharge; therefore, inverse causality (i.e., infectious diseases and health care costs influencing type of feeding) cannot be excluded regarding data at 2, 4, 6, 9 and 12 months. Our study has some strengths too. Firstly, it is based on a cohort recruited in the main hospital in Cantabria (Spain), were more than 90% births in the region took place. Secondly, most studies on type of feeding costs are simulations [8,16,21,24], not actual cohorts. Simulation studies are important for generalizing, but they are based on indirect data and so are prone to bias in the extrapolation process.

Summarizing, our results indicate that health care costs due to infectious diseases in neonates fed with artificial feeding were about 900€ per child in the first year of life. As 18% of neonates in our cohort were fed with artificial feeding at hospital discharge, further efforts should be made to increase both initiation and continuation of breastfeeding.

## Figures and Tables

**Table 1:** Main characteristics of participants in the study.

Pregnant Women	*n* = 948	%	Range
Maternal age in years. Mean and standard deviation	33.7	5.2	17–52
Twin gestation			
No	926	97.7	
Yes	22	2.3	
Maternal smoking			
No	830	87.6	
Yes	118	12.5	
Cigarettes/day. Mean and standard deviation	7.2	5.3	
Maternal educational level			
Primary studies	214	22.6	
Secondary studies	111	11.7	
Foundation degree	273	28.8	
University studies	350	36.9	
Occupational status			
Working	660	69.6	
Unemployed	162	17.1	
No active	116	12.2	
Student	10	1.1	
**Neonates**	***n*** **= 970**	**%**	**Range**
Gender			
Male	490	50.5	
Female	480	49.5	
Pregnancy length in weeks. Mean and standard deviation	39.1	2.0	25–42
≥37 weeks	910	93.8	
34–36 weeks	39	4.0	
<34 weeks	21	2.2	
Birthweight in grams. Mean and standard deviation	3244.5	572.3	870–4840
2500–4000 g	806	83.1	
>4000 g	80	8.3	
<2500 g	84	8.7	
Feeding at hospital discharge			
Exclusive breastfeeding	524	54.0	
Mixed breastfeeding + artificial feeding	272	28.0	
Artificial feeding	174	17.9	
Nursery attendance			
No	763	78.7	
Yes	132	13.6	
Unknown	74	7.6	

**Table 2:** Relationship between type of feeding and costs due to consultations in primary care.

Time	Type of Feeding	*n* Consultations/*n* Neonates *	Average Cost (€) **	95% CI **		*p* **
Hospital discharge	Exclusive breastfeeding	1217/524	197.9	177.0	218.8	
	Mixed breastfeeding and artificial	744/272	223.2	194.8	251.6	0.17
	Artificial	683/174	295.7	258.5	332.8	<0.001
2 months	Exclusive breastfeeding	1032/427	173.4	149.4	197.4	
	Mixed breastfeeding and artificial	499/183	223.8	188.2	259.4	0.02
	Artificial	1124/299	257.8	228.5	287.0	<0.001
	Missing	134				
4 months	Exclusive breastfeeding	815/354	166.2	139.5	192.9	
	Mixed breastfeeding and artificial	442/164	222.3	184.4	260.2	0.02
	Artificial	1398/387	249.0	222.5	275.6	<0.001
	Missing	181				
6 months	Exclusive breastfeeding	531/238	141.3	110.4	172.3	-
	Mixed breastfeeding and artificial	467/183	155.7	121.0	190.3	0.53
	Artificial	1657/483	206.2	182.8	229.7	<0.001
	Missing	66				
9 months	Exclusive breastfeeding	0				
	Mixed breastfeeding and artificial	759/318	170.9	142.7	199.1	
	Artificial	1874/573	221.5	199.2	243.8	0.004
	Lost to follow-up	79				
12 months	Exclusive breastfeeding	0				
	Mixed breastfeeding and artificial	553/239	180.1	147.8	212.4	
	Artificial	2060/642	226.6	205.8	247.3	0.01
	Missing	89				

* Total number of neonates does not add up to 970 due to missing data in the follow-up. ** Marginal means adjusted for maternal smoking, maternal educational level, maternal occupational status, twin pregnancy, gestation length, birth order, nursery attendance (yes/no) and age of starting nursery attendance.

**Table 3:** Relationship between type of feeding and costs due to consultations in emergency room.

Time	Type of Feeding	*n* Consultations in ER/*n* Neonates	Average Cost (€) *	95% CI *		*p* *
Hospital discharge	Exclusive breastfeeding	543/524	196.2	165.3	227.0	
	Mixed breastfeeding and artificial	362/272	212.8	171.3	254.3	0.53
	Artificial	276/174	260.1	206.7	313.6	0.05
2 months	Exclusive breastfeeding	441/427	181.8	148.6	215.0	
	Mixed breastfeeding and artificial	187/183	170.4	120.7	220.2	0.71
	Artificial	537/299	281.0	241.1	320.8	<0.001
4 months	Exclusive breastfeeding	335/354	170.2	133.6	206.8	
	Mixed breastfeeding and artificial	178/164	182.5	130.0	235.0	0.71
	Artificial	652/387	264.3	229.1	299.5	<0.001
6 months	Exclusive breastfeeding	219/238	164.2	119.8	208.7	
	Mixed breastfeeding and artificial	180/183	173.8	124.2	223.4	0.78
	Artificial	764/483	250.4	219.1	281.8	0.002
9 months	Exclusive breastfeeding	0				
	Mixed breastfeeding and artificial	299/318	171.0	132.7	209.3	
	Artificial	855/573	240.9	212.2	269.6	0.005
12 months	Exclusive breastfeeding	0				
	Mixed breastfeeding and artificial	228/239	175.7	131.4	220.0	
	Artificial	925/642	235.8	208.8	262.9	0.02

* Total number of neonates does not add up to 970 due to missing data in the follow-up. ** Marginal means adjusted for maternal smoking, maternal educational level, maternal occupational status, twin pregnancy, gestation length, birth order, nursery attendance (yes/no) and age of starting nursery attendance.

**Table 4:** Relationship between type of feeding and costs due to admissions for infectious disease.

Time	Type of Feeding	*n* Admissions/*n* Neonates	Average Cost (€) *	95% CI *		*p* *
Hospital discharge	Exclusive breastfeeding	7/524	86.9		328.8	
	Mixed breastfeeding and artificial	17/272	166.5		491.0	0.70
	Artificial	15/174	791.6	372.9	1210	0.005
2 months	Exclusive breastfeeding	7/427	152.4		416.0	
	Mixed breastfeeding and artificial	5/183	94.1		416.0	0.81
	Artificial	58/299	424.0	107.2	740.8	0.21
4 months	Exclusive breastfeeding	4/354	126.6		416.7	
	Mixed breastfeeding and artificial	7/164	93.8		510.4	0.90
	Artificial	26/387	383.8	104.4	663.1	0.22
6 months	Exclusive breastfeeding	2/238	85.6		438.1	-
	Mixed breastfeeding and artificial	7/183	136.9		529.9	0.85
	Artificial	28/483	330.4	82.0	578.8	0.85
9 months	Exclusive breastfeeding	0				
	Mixed breastfeeding and artificial	4/318	163.9		467.6	
	Artificial	32/573	286.8	59.4	514.3	0.53
12 months	Exclusive breastfeeding	0				
	Mixed breastfeeding and artificial	4/239	163.9		514.3	
	Artificial	33/642	279.5	65.5	493.5	0.58

* Total number of neonates does not add up to 970 due to missing data in the follow-up. ** Marginal means adjusted for maternal smoking, maternal educational level, maternal occupational status, twin pregnancy, gestation length, birth order, nursery attendance (yes/no) and age of starting nursery attendance.

**Table 5:** Relationship between type of feeding and all costs due to infectious diseases.

Time	Type of Feeding	*n*	Average Cost (€) *	95% CI *		*p* *
Hospital discharge	Exclusive breastfeeding	524	443.5	193.7	694.0	
	Mixed breastfeeding and artificial	272	571.4	235.0	907.8	0.56
	Artificial	174	1339.5	903.0	1775.0	0.001
2 months	Exclusive breastfeeding	427	480.3	207.5	753.1	
	Mixed breastfeeding and artificial	183	463.3	54.2	872.4	0.95
	Artificial	299	948.5	619.8	1277.2	0.04
4 months	Exclusive breastfeeding	354	435.4	135.0	735.8	
	Mixed breastfeeding and artificial	164	476.9	46.0	907.7	0.88
	Artificial	387	881.1	591.7	1170.6	0.04
6 months	Exclusive breastfeeding	238	378.8	14.3	743.3	
	Mixed breastfeeding and artificial	183	466.2	58.7	873.7	0.75
	Artificial	483	790.1	532.9	1047.3	0.08
9 months	Exclusive breastfeeding	0				
	Mixed breastfeeding and artificial	318	482.0	167.8	796.3	
	Artificial	573	734.3	498.5	970.1	0.21
12 months	Exclusive breastfeeding	0				
	Mixed breastfeeding and artificial	239	491.4	128.2	854.7	
	Artificial	642	720.2	498.4	942.1	0.30

* Total number of neonates does not add up to 970 due to missing data in the follow-up.** Marginal means adjusted for maternal smoking, maternal educational level, maternal occupational status, twin pregnancy, gestation length, birth order, nursery attendance (yes/no) and age of starting nursery attendance.

## Supplementary Materials

The following are available online at https://www.mdpi.com/1660-4601/17/13/4719/s1, Table S1: Admissions to hospital in the first year of life. All patient refined—Diagnosis Related Groups, severity, weight, and cost, Table S2: Relationship between type of feeding and costs due to treatment with drugs.
