# Supplementary material for: Health Care Costs Associated to Type of Feeding in the First Year of Life
Source: Int J Environ Res Public Health. 2020 Jun 30;17(13):4719. doi: 10.3390/ijerph17134719 (PMC7369965; doi:10.3390/ijerph17134719)
Supplement: Supplementary file 1 [file ijerph-17-04719-s001.pdf]

International Journal of
Environmental Research and Public Health

MDPI

Supplementary Table S1. Admissions to hospital in the first year of life. All patient refined—Diagnosis Related Groups, severity, weight, and cost.

APR-DRG *	Description	Severity	Weight **	Normalized Cost (€/Patient) ***	Number of Patients	Total Cost ****
113	Infections of upper respiratory tract	1	0.2701	1357.4094	4	5429.6376
138	RSV pneumonia	1	0.2731	1372.4862	10	13,724.862
139	Other pneumonia	1	0.39	1959.9766	1	1959.9766
141	Bronchiolitis and asthma	1	0.345	1733.8254	3	5201.4762
141	Bronchiolitis and asthma	2	0.51	2563.0463	1	2563.0463
144	Respiratory signs, symptoms and minor diagnoses	1	0.4281	2151.4512	2	4301.9024
249	Non-bacterial gastroenteritis, nausea and vomiting	1	0.3694	1856.4496	3	5569.3488
249	Non-bacterial gastroenteritis, nausea and vomiting	2	0.48	2412.28	2	4824.56
385	Other skin, subcutaneous tissue and breast disorders	1	0.3755	1887.1057	1	1887.1057
463	Kidney and urinary tract infections	1	0.3999	2009.7298	1	2009.7298
602	Neonate birthweight 1000–1249 g with respiratory distress syndrome / other major respiratory or major anomaly	3	8.7849	44,149.227	1	44,149.227
609	Neonate birthweight 1500–2499 g with major procedure	2	14.0684	70,701.884	1	70,701.884
611	Neonate birthweight 1500–1999 g with major anomaly	1	2.9476	14,813.403	1	14,813.403
640	Neonate birthweight >2499 g, normal newborn or neonate with other problem	2	0.1371	689.00716	1	689.00716
710	Infectious & parasitic diseases including HIV with O.R. procedure	1	1.0636	5345.208	1	5345.208
722	Fever	1	0.3436	1726.7896	1	1726.7896
723	Viral illness	1	0.3265	1640.8522	3	4922.5566
724	Other infectious and parasitic diseases	1	0.5869	2949.5135	4	11,798.054
724	Other infectious and parasitic diseases	2	0.7139	3587.7623	1	3587.7623
861	Signs, symptoms and other factors influencing health status	1	0.3815	1917.2588	1	1917.2588

* APR–DRG: All patients refined—Diagnosis Related Groups. ** Weight—according to the Spanish Norm for APR-GDRs [26]. *** Normalized cost = weight × 5025.58€ (according to the Spanish Norm for APR-GDRs[26]). **** Total cost = normalized cost × number of patients.

Supplementary Table S2. Relationship between type of feeding and costs due to treatment with drugs.

Time	Type of Feeding	*n*	Average Cost (€) *	95% CI*		*p* *
Hospital Discharge	Exclusive breastfeeding	524	6.64	5.86	7.42	
	Mixed breastfeeding and artificial	272	6.55	5.51	7.60	0.90
	Artificial	174	8.81	7.46	10.17	0.007
2 Months	Exclusive breastfeeding	427	6.32	5.48	7.16	
	Mixed breastfeeding and artificial	183	6.84	5.58	8.10	0.50
	Artificial	299	8.07	7.06	9.09	0.01
4 Months	Exclusive breastfeeding	354	5.92	4.99	6.84	
	Mixed breastfeeding and artificial	164	6.70	5.37	8.03	0.34
	Artificial	387	8.14	7.25	9.04	0.001
6 Months	Exclusive breastfeeding	238	5.71	4.60	6.84	
	Mixed breastfeeding and artificial	183	6.22	4.96	7.47	0.56
	Artificial	483	7.96	7.17	8.75	0.002
9 Months	Exclusive breastfeeding	0				
	Mixed breastfeeding and artificial	318	5.86	4.89	6.83	
	Artificial	573	7.76	7.04	8.49	0.002
12 Months	Exclusive breastfeeding	0				
	Mixed breastfeeding and artificial	239	5.72	4.60	6.84	
	Artificial	642	7.64	6.95	8.32	0.004

* Total number of neonates does not add to 770 due to missing data in the follow-up. ** Marginal means adjusted for maternal smoking, maternal educational level, maternal occupational status, twin pregnancy, gestation length, birth order, nursery attendance (yes/no) and age of starting nursery attendance.
